# Toward Biology-Driven Surveillance After Endometrial Cancer Treatment: A Molecular–Clinical Framework Integrating Recurrence Phenotype

**DOI:** 10.3390/cancers18091443

**Published:** 2026-04-30

**Authors:** Wiktor Szatkowski, Izabela Glanowska-Nawrat

**Affiliations:** 1 Department of Gynecologic Oncology, Maria Skłodowska-Curie National Research Institute of Oncology, 31-115 Kraków, Poland; 2 Department of Oncology, Zealand University Hospital, 4700 Naestved, Denmark; iglanowska@hotmail.com

**Keywords:** endometrial cancer, surveillance, follow-up, molecular classification, recurrence patterns, immunotherapy, CTNNB1, beta-catenin, L1CAM, precision oncology

## Abstract

**Background:** Current post-treatment surveillance strategies for endometrial cancer rely predominantly on clinical stage and histological grade, without integration of molecular tumor biology. Molecular classification has revealed profound biological heterogeneity across endometrial cancer subtypes, including differences in recurrence patterns, prognosis, and treatment responsiveness, yet surveillance strategies have not been systematically adapted to reflect this heterogeneity. **Objective:** To propose a biology-driven surveillance framework for endometrial cancer that integrates molecular subtype, clinicopathological risk factors, and recurrence phenotype. **Methods:** This narrative review and conceptual framework synthesizes evidence from cohort studies, molecular classification analyses, international guidelines, and the literature addressing recurrence patterns and treatment responsiveness across molecular subtypes of endometrial cancer. **Results:** We propose a three-tier surveillance model stratifying patients into low-, intermediate-, and high-risk groups. The framework integrates molecular subtype with clinicopathological modifiers and expected recurrence phenotype. Within the no specific molecular profile (NSMP) subtype, CTNNB1 mutation status is incorporated as a primary modifier, assigning CTNNB1-mutated tumors to the intermediate-risk group regardless of estrogen receptor (ER) status. In CTNNB1 wild-type NSMP tumors, ER expression functions as a secondary modifier, allowing identification of a biologically low-risk subgroup. L1CAM expression is considered a high-risk modifier within NSMP. The framework also accounts for differences in the therapeutic modifiability of recurrence, including the role of immunotherapy in mismatch repair-deficient tumors. **Conclusions:** Uniform post-treatment surveillance does not reflect the biological diversity of endometrial cancer. The proposed framework provides a biologically grounded approach to surveillance that aligns follow-up intensity with recurrence phenotype and therapeutic opportunities. This model may serve as a conceptual basis for prospective studies evaluating personalized surveillance strategies in endometrial cancer.

## Simple Summary

Follow-up after endometrial cancer treatment is still largely based on clinical stage and histological features, often applying similar visit schedules to biologically distinct tumors. However, molecular classification has shown that endometrial cancer subtypes differ in prognosis, recurrence pattern, and treatment options after relapse. In this paper, we propose a biology-driven post-treatment surveillance framework that combines molecular subtype, clinicopathological factors, and expected recurrence phenotype. The model is based on a simple principle: surveillance should be most intensive when earlier detection of recurrence is most likely to change treatment. This approach may help reduce unnecessary surveillance in biologically low-risk disease while supporting closer monitoring in patients whose recurrence may remain treatable. The proposed framework is conceptual and requires prospective validation.

## 1. Introduction

Endometrial cancer (EC) is the most common gynecologic malignancy in developed countries [1,2]. Despite the favorable prognosis observed in the majority of patients, disease recurrence remains a clinically significant problem that determines the ultimate treatment outcome and limits therapeutic options. The rising incidence observed over recent decades, driven in part by the increasing prevalence of obesity and type 2 diabetes, means that optimizing post-treatment management is becoming increasingly important from both clinical and health-economic perspectives [3].

The traditional approach to post-treatment surveillance after endometrial cancer treatment originates from the pre-molecular era and relies primarily on FIGO clinical stage and histological grade. For many years, the absence of randomized clinical trial data resulted in considerable heterogeneity in surveillance schedules, both in terms of visit frequency and the scope of investigations performed. A landmark attempt to address this gap was the TOTEM trial, the largest randomized study to date evaluating follow-up intensity after endometrial cancer treatment. Conducted across 42 centers in Italy and France, it enrolled 1847 patients [4]. After a median follow-up of 69 months, five-year overall survival was 90.6% in the intensive surveillance arm and 91.9% in the minimalist arm (HR 1.13; 95% CI 0.86–1.50), with no significant difference in any of the analyzed subgroups. The study also showed that intensive surveillance did not improve quality of life, while providing no support for a more resource-intensive surveillance strategy [4,5]. The results of the TOTEM trial informed changes in the guidelines of numerous scientific societies.

However, the TOTEM trial was designed and conducted before the era of molecular classification of endometrial cancer. Risk stratification was based exclusively on clinical and histological criteria, without incorporating biological tumor subtype. In the past decade, however, a fundamental shift has occurred in the understanding of endometrial cancer biology, driven by the implementation of genomic classification from The Cancer Genome Atlas (TCGA) [6]. This system identifies four molecular subtypes: POLE-mutated (POLEmut), mismatch repair-deficient (MMRd), p53-aberrant (p53abn), and no specific molecular profile (NSMP). These subtypes differ profoundly not only in prognosis, but also in recurrence dynamics, relapse site, and responsiveness to systemic treatment. Patients with the POLEmut subtype have an exceptionally favorable prognosis with near-absent recurrence, while the p53abn subtype is associated with recurrence and disease-related mortality exceeding 30% [6].

The integration of molecular classification into clinical practice has been reflected in updated guidelines. In 2023, a revised FIGO staging system was published incorporating tumor biological factors—including molecular classification—alongside traditional anatomical factors [7]. In response, the ESGO, ESTRO, and ESP updated their joint guidelines on the management of endometrial cancer [8]. Despite this progress, the implications of molecular classification for post-treatment surveillance strategies remain insufficiently defined. Current surveillance recommendations still do not adequately differentiate surveillance schedules according to molecular tumor subtype.

A key argument for individualizing surveillance is the heterogeneity of recurrence phenotypes across molecular subtypes. Isolated vaginal and nodal recurrences, characteristic of the MMRd and NSMP subtypes, may be amenable to treatment with curative intent—both surgical and salvage radiotherapy—and, in the case of MMRd, immunotherapy based on immune checkpoint inhibitors. In stark contrast, peritoneal and distant recurrences, typical of the p53abn subtype, are usually systemic and unresectable, limiting the impact of early detection on treatment outcome. The value of post-treatment surveillance should therefore be assessed not by the frequency of detected recurrences per se, but by the potential for meaningful modification of subsequent therapeutic management following their identification.

The aim of this paper is to propose a biologically grounded surveillance framework after endometrial cancer treatment that integrates molecular subtype, clinical risk factors, and the expected phenotype and therapeutic modifiability of potential recurrence. The proposed model is based on a synthesis of published cohort data, multicenter analyses, current international guidelines, and available evidence on recurrence treatment across molecular subtypes.

It is worth noting that some national healthcare systems are already evolving toward individualized surveillance. A notable example is the Danish national guideline (Danish Centre for Health Technology Assessment), which introduced risk-based surveillance stratification, selective use of imaging, and a strong emphasis on patient education and clinical examination as the cornerstone of surveillance [9]. Although this guideline does not yet incorporate molecular classification, its organizational structure provides a practical proof of concept for stratified surveillance models—a concept that the proposed framework extends with a biological dimension.

## 2. Scope and Methodological Approach

This manuscript is a narrative review and conceptual framework paper. It does not present original research data and was not designed as a systematic review. Instead, it provides a focused, biologically oriented synthesis of the literature to support the development of a molecular–clinical surveillance model after endometrial cancer treatment.

The proposed framework was developed through an interpretive review of published evidence addressing: (1) molecular classification of endometrial cancer, (2) recurrence patterns across molecular subtypes, (3) clinicopathological modifiers of recurrence risk, (4) the therapeutic modifiability of recurrence, and (5) international recommendations on post-treatment surveillance.

We searched PubMed/MEDLINE and guideline documents from major societies (ESGO, ESMO, NCCN, FIGO) for studies published primarily between 2015 and 2025 using combinations of terms such as “endometrial cancer”, “surveillance”, “follow-up”, “molecular classification”, “recurrence pattern”, “MMRd”, “POLE”, “p53”, “NSMP”, “CTNNB1”, “L1CAM”, and “immunotherapy”. Additional manual searches of reference lists of relevant articles were also performed.

This was a targeted, non-systematic search intended to support a biologically oriented synthesis rather than a comprehensive systematic review. Studies were included if they addressed recurrence patterns, post-recurrence outcomes, or surveillance implications in molecularly defined endometrial cancer. Studies lacking molecular classification data or not reporting clinically relevant recurrence characteristics were not considered in the primary synthesis. Priority was given to molecularly annotated cohort studies, clinically relevant translational analyses, randomized trials with subgroup data, and contemporary international guidelines. Case reports, small non-comparative series, and studies not addressing recurrence phenotype, surveillance implications, or therapeutic modifiability were used only as contextual support.

The underlying conceptual assumption of this work is that the clinical value of surveillance is greatest when earlier detection of recurrence may alter management, particularly by enabling potentially curative local treatment or timely use of biologically informed systemic therapy. On this basis, the proposed model links surveillance intensity not only to baseline recurrence risk, but also to expected recurrence phenotype and therapeutic opportunity.

Given the narrative nature of this review, evidence was not formally graded; however, a hierarchical approach to evidence interpretation was applied. Randomized controlled trials and their molecular subgroup analyses were considered the highest level of evidence, followed by large prospective or retrospective molecularly annotated cohort studies. Guideline-based and consensus data were used to provide contextual support. Conclusions derived from higher-level evidence are presented with greater confidence, whereas findings from smaller or exploratory studies are interpreted with appropriate caution.

Because this framework has not been prospectively validated, it should be considered hypothesis-generating and intended as a basis for future prospective and health-economic evaluation. To date, no randomized controlled trial has evaluated molecularly stratified post-treatment surveillance strategies in endometrial cancer; the proposed framework therefore represents a conceptual model rather than an evidence-based protocol.

### 2.1. Molecular Classification and Recurrence Risk

The foundation of the proposed model is the profound biological heterogeneity of endometrial cancer revealed by TCGA genomic classification and its clinical adaptation. The four recognized molecular subtypes—POLEmut, MMRd, p53abn, and NSMP—differ not only in prognosis but, more importantly, in recurrence pattern, relapse site, and susceptibility to treatment, all of which have direct implications for post-treatment surveillance strategies [10,11,12]. Table 1 presents a molecular subtype-based stratification integrating recurrence risk and surveillance implications, while Table 2 provides a complementary phenotype-oriented clinical perspective.

The POLEmut subtype has an exceptionally favorable clinical course. Multiple retrospective studies have demonstrated that five-year recurrence-free survival (RFS) approaches 100% in these patients, regardless of clinical stage [13]. Data from the PORTEC-3 trial, enrolling 410 patients with high clinical risk, confirmed excellent treatment outcomes in POLEmut patients—five-year RFS was 98% in both trial arms, irrespective of adjuvant chemotherapy use [14,15]. The exceptionally low recurrence risk justifies substantial de-escalation of surveillance intensity, and routine imaging in this group has no biological justification.

The MMRd subtype is characterized by a distinctive recurrence pattern with a predominance of locoregional relapse. The KImBer cohort analysis, encompassing 101 patients with endometrial cancer recurrence, demonstrated that locoregional recurrences accounted for 46% of all relapses in the MMRd group, compared with only 20% in the p53abn group, and that isolated vaginal recurrences occurred in 36% of MMRd patients versus 10% in the p53abn group [16]. Median post-recurrence survival was most favorable for MMRd patients (43 months), compared with 39 months for NSMP and only 10 months for p53abn (*p* = 0.001) [16]. The clinical and pathological characteristics associated with the MMRd subtype have been further described in large cooperative group analyses [17]. Furthermore, the MMRd subtype is characterized by high sensitivity to immune checkpoint inhibitors, which further increases the clinical value of early recurrence detection.

The p53abn subtype exhibits a markedly different and unfavorable recurrence pattern. In the KImBer cohort, patients with p53abn most frequently experienced abdominal recurrences (43%) compared with the MMRd group (18%) and NSMP group (31%), with non-locoregional relapses accounting for 80% of all recurrences [16]. Data from a Danish study that focused on high-grade stage I tumors confirm this pattern: five-year overall recurrence rates were 35% for p53abn, with abdominal and distant relapses predominating over locoregional recurrences [18]. The short post-recurrence survival—a median of only 10 months—and the low response rate to conventional chemotherapy (objective response rate, ORR 20–30%) limit the practical benefit of early detection in this group. The primary surveillance goal in the p53abn subtype should be prompt initiation of systemic therapy or enrollment in clinical trials.

The NSMP subtype is a biologically heterogeneous group. Low-grade tumors (G1–2) without additional risk factors exhibit a recurrence pattern similar to MMRd, with predominant locoregional relapse and relatively favorable post-recurrence prognosis. In contrast, high-grade NSMP (G3) or tumors with substantial LVSI behave more aggressively, with a higher proportion of distant and abdominal relapses, making them biologically closer to the p53abn subtype. This internal heterogeneity of the NSMP group requires the incorporation of additional clinicopathological factors when assigning patients to risk groups. Subtype-specific recurrence patterns, timing, and clinical implications are summarized in Table 1.

**Table 1:** Molecular subtype-specific recurrence patterns, post-recurrence outcomes, and surveillance tier assignment in endometrial cancer.

Molecular Subtype	Recurrence Risk	Dominant Pattern & Time to Recurrence	OS After Recurrence	Clinical Value of Early Detection & Surveillance Tier
**POLEmut** [6,13,14,15]	**Very low (<5%)**	Rare; if present, isolated locoregional. Time to recurrence not well-defined (rare events).	Excellent; near-normal OS	VERY LOW—de-escalation justified; routine imaging not indicated. **→ LOW**
**MMRd** [16,19,20,21]	**Intermediate (variable across cohorts)**	Predominantly locoregional: vaginal (~36%), pelvic/nodal (~46%). Median ~16 months.	Favorable (~43 months median)	HIGH—salvage RT feasible; strong rationale for PD-1 inhibitors; dual benefit of early detection. **→ INTERMEDIATE**
**p53abn** [16,18]	**High (~30–35%; stage I high-grade cohorts)**	Predominantly distant/peritoneal (~80% non-locoregional). Median ~12–18 months.	Poor (~10 months median)	LOW—early detection rarely enables curative intent; priority: rapid systemic therapy or trial enrollment. **→ HIGH**
**NSMP (overall)** [16,22,23]	**Low–intermediate (~10–20%)**	Predominantly locoregional; higher-grade may recur distantly. Median ~16 months.	Intermediate (~39 months median)	MODERATE—value depends on modifiers (CTNNB1, ER, L1CAM, grade). See subgroups below. **→ See subgroups**
NSMP, CTNNB1mut	Intermediate–higher	Increased nodal and early locoregional risk. Time: variable.	Potentially less favorable than CTNNB1wt	MODERATE–HIGH—clinically actionable recurrence potential; supports closer follow-up. **→ INTERMEDIATE**
NSMP, ER+, CTNNB1wt, L1CAM−	Lower within NSMP	Predominantly locoregional; biologically indolent. Time: variable.	More favorable	MODERATE—may support de-escalation toward low-risk surveillance intensity. **→ LOW**
NSMP, ER− or L1CAM+	Higher within NSMP	Increased distant recurrence risk; more aggressive phenotype. Time: variable.	Less favorable	MODERATE–LOW—lower imaging threshold; consider higher-risk classification. **→ INTERMEDIATE/HIGH**

Recurrence risk estimates are approximate and derived from heterogeneous cohort studies; survival data should be interpreted with caution. Median OS after recurrence for MMRd (~43 months) and p53abn (~10 months) from the KImBer cohort [16]. p53abn recurrence rate (~30–35%) from stage I high-grade cohort [18]. NSMP subgroups are conceptual stratification pathways pending prospective validation; CTNNB1 as surveillance modifier is a candidate criterion. OS—overall survival; MMRd—mismatch repair-deficient; POLEmut—pathogenic POLE mutation; ER—estrogen receptor; L1CAM—L1 cell adhesion molecule; CTNNB1—β-catenin gene; PD-1—programmed death-1; RT—radiotherapy.

**Table 2:** Recurrence phenotypes in endometrial cancer: anatomical distribution, potential for curative treatment, and implications for surveillance strategy.

Recurrence Phenotype	Typical Location	Potential for Curative Treatment	Implications for Surveillance Strategy	Key Survival Data
**Isolated vaginal recurrence**	Vaginal vault	**HIGH**	Early detection has direct clinical value; enables salvage radiotherapy ± surgery with curative intent. Strong justification for intensive clinical examination in groups where this phenotype is expected (MMRd, low-risk NSMP).	5-yr OS 70–83% after salvage RT [24,25]
**Isolated pelvic recurrence**	Parametria, pelvic sidewall	**MODERATE**	Clinical examination (bimanual) has high detection value. Pelvic MRI indicated when clinically suspected. Radical RT or surgery feasible in selected cases.	Favorable if isolated; cohort data [24]
**Isolated nodal recurrence**	Pelvic/para-aortic lymph nodes	**MODERATE–HIGH**	Multimodality treatment (resection ± RT ± chemotherapy) may achieve curative intent if isolated. Prerequisite: absence of peritoneal or distant spread.	2-yr PFS 50–60% with multimodality treatment [26,27]
**Oligometastatic recurrence**	Lung, liver, or bone (isolated lesions)	**LIMITED**	SBRT/SABR or surgical resection may achieve durable remission in carefully selected patients with prolonged disease-free interval. Primarily MMRd and NSMP subtypes.	Retrospective series; no RCT data
**Peritoneal/multifocal recurrence**	Peritoneum, abdominal cavity	**LOW**	Systemic therapy is the standard of care. Early imaging detection rarely changes management. Surveillance goal: prompt eligibility assessment for systemic therapy or clinical trials.	Median OS~10–12 months (p53abn) [16]
**Systemic/disseminated recurrence**	Multiorgan	**VERY LOW**	Priority: rapid initiation of systemic therapy. Surveillance should focus on symptom recognition and performance status monitoring rather than asymptomatic imaging detection.	Median OS < 12 months [16]

Color coding: green—high or moderate potential for curative-intent treatment; orange—limited; red—low curative potential. Table 1 presents a molecular subtype-based stratification integrating recurrence risk and surveillance implications; Table 2 provides a phenotype-oriented clinical perspective. Survival data are derived from heterogeneous sources including cohort studies and retrospective analyses; direct comparisons should be interpreted with caution. OS—overall survival; RT—radiotherapy; SBRT/SABR—stereotactic body/ablative radiotherapy; PFS—progression-free survival; RCT—randomized controlled trial.

### 2.2. Time to Recurrence and the Therapeutic Window

Regardless of molecular subtype, the majority of endometrial cancer recurrences occur within the first two years after primary treatment. The median time to first recurrence was 16 months across the entire cohort, with no statistically significant differences between subtypes [16]. Surveillance in the first two years after treatment has the greatest clinical justification and should be most intensive. At the same time, late recurrences—beyond five years—were observed almost exclusively in the MMRd and NSMP subtypes, supporting the continuation of surveillance in these groups beyond the standard five-year period.

### 2.3. Implications for the Surveillance Goal

The described differences in recurrence biology lead to a practical conclusion that underpins the proposed framework: not all recurrences carry equal clinical value from a surveillance perspective. Locoregional relapses are potentially curable, and their early detection has a direct therapeutic impact. Peritoneal relapses typical of the p53abn subtype rarely qualify for local treatment. Surveillance strategies should therefore be guided by the question of whether detecting a given recurrence changes subsequent clinical management. This distinction provides the conceptual basis for a surveillance model that prioritizes clinically actionable recurrence detection over uniform visit intensity.

## 3. Recurrence Phenotype and Therapeutic Implications

The pattern of endometrial cancer recurrence carries fundamental significance not only in terms of prognosis but, more importantly, in terms of therapeutic management. The feasibility of treatment with curative intent following recurrence is critically dependent on its phenotype: the site, extent, and biology of the relapse. Not every recurrence is clinically equivalent from the standpoint of therapeutic modifiability.

Isolated vaginal recurrence represents the paradigmatic example of a potentially curable relapse. Retrospective data demonstrate that salvage radiotherapy achieves five-year overall survival of 83%, with locoregional control of 89% and regional control of 91.5% [24]. Independent predictors of a favorable outcome include an isolated vaginal site and a recurrence interval of more than nine months from completion of primary treatment. Data from the PORTEC-1 trial analysis indicate that in patients without adjuvant radiotherapy who developed vaginal recurrence, five-year overall survival after salvage treatment was 70%, with a complete response rate to salvage radiotherapy of 89% [25]. These results provide direct justification for intensive clinical surveillance in risk groups where isolated vaginal recurrence is the biologically expected phenotype—primarily the MMRd and NSMP subtypes.

Isolated nodal recurrences—pelvic and para-aortic—represent a second category amenable to treatment with curative intent. Multimodality treatment of nodal recurrences, incorporating cytoreductive surgery, radiotherapy, and chemotherapy, can achieve two-year progression-free survival rates of 50–60% [26,27], although data from randomized trials in this area remain limited. The key criterion is an isolated recurrence without concurrent peritoneal dissemination or distant metastases.

Oligometastatic recurrences—isolated pulmonary, hepatic, or bone metastases—represent a distinct category. Although prognosis in this group is less favorable than with locoregional recurrences, in carefully selected cases, stereotactic body/ablative radiotherapy (SBRT/SABR) or surgical resection may lead to durable remission, particularly when the disease-free interval is prolonged. This applies primarily to the MMRd subtype and selected NSMP patients.

In marked contrast, peritoneal, multifocal, and disseminated recurrences—characteristic of the p53abn subtype—rarely qualify for local treatment. Systemic therapy in this group is primarily palliative in intent. For patients with advanced or recurrent endometrial cancer of the p53abn subtype treated with platinum–taxane chemotherapy, median progression-free survival is below six months, and median overall survival is approximately 12 months. In this group, early detection of recurrence—unless it alters therapeutic options—has limited impact on ultimate prognosis. These observations support the concept that surveillance strategies should be aligned not only with recurrence risk, but also with the likelihood that recurrence will remain amenable to clinically meaningful intervention.

The detailed characterization of individual recurrence phenotypes, their potential for curative treatment, and implications for surveillance strategies are presented in Table 2.

## 4. Proposed Surveillance Stratification Model

### 4.1. Rationale for the Three-Tier Model

Based on the relationships described above between molecular subtypes, recurrence patterns, and therapeutic potential, we propose a three-tier surveillance model that assigns patients to one of three groups: low, intermediate, and high risk. This model is not intended as a prognostic classification system; rather, its purpose is to provide a biologically informed approach to stratifying surveillance intensity in a clinically useful manner.

It should be noted that the updated ESGO–ESTRO–ESP 2025 guidelines distinguish four prognostic groups: low, intermediate, high–intermediate, and high risk. In the proposed surveillance model, the intermediate and high–intermediate groups according to ESGO 2025 may reasonably be merged into a single intermediate surveillance category, on the grounds of broadly similar recurrence phenotype (locoregional predominance, therapeutic potential) and a comparable rationale for clinical surveillance intensity. This pragmatic simplification is distinct from adjuvant treatment stratification, for which the distinction between these subgroups remains clinically important.

The key conceptual feature distinguishing the proposed model from existing surveillance strategies is that surveillance intensity is determined not solely by baseline recurrence risk, but by the expected recurrence phenotype and the anticipated potential for therapeutic modification. This means that some patients with high biological recurrence risk (e.g., p53abn at advanced stage) may require intensive clinical surveillance aimed primarily at facilitating rapid initiation of systemic therapy, rather than routine intensive imaging. Conversely, patients in biologically intermediate groups (MMRd, NSMP) may justify heightened clinical vigilance precisely because their recurrences may remain amenable to curative-intent treatment.

### 4.2. Proposed Risk Group Assignment Criteria

Within the low-risk group, two broad patient categories may be considered. The first includes all patients with the POLEmut subtype, irrespective of clinical stage and histological grade, reflecting the consistently observed, exceptionally low recurrence risk in this subgroup. The second category encompasses patients with the NSMP subtype who simultaneously fulfill low-risk clinicopathological criteria—stage I, G1–2, myometrial invasion < 50%, absent/focal LVSI, pN0—and demonstrate the absence of CTNNB1 mutation, positive estrogen receptor (ER) expression, and the absence of L1CAM overexpression. The inclusion of CTNNB1, ER, and L1CAM as modifiers is proposed exclusively within the NSMP group and reflects its biological heterogeneity. This profile may correspond to the most biologically indolent form of NSMP and may justify de-escalation of surveillance intensity.

Within the intermediate-risk group, surveillance may carry the greatest clinical value, as the expected recurrence phenotype is more frequently locoregional or nodal—and therefore potentially amenable to local treatment and, in the MMRd subtype, also to immunotherapy. This group may include: (i) patients with the MMRd subtype who do not meet high-risk criteria; (ii) patients with NSMP and intermediate clinicopathological risk factors; (iii) patients with NSMP and CTNNB1 mutation, irrespective of ER status, who otherwise fulfill low-risk anatomical criteria; and (iv) patients with ER-negative NSMP in the absence of CTNNB1 mutation or without CTNNB1 assessment.

Assignment of CTNNB1-mutated NSMP tumors to the intermediate-risk group reflects emerging evidence that this subgroup deviates from the biologically indolent NSMP phenotype despite otherwise favorable clinicopathological features. Several studies suggest that CTNNB1 mutations are associated with an increased risk of recurrence, including early and locoregional relapse, even in low-stage disease. Although these findings are not entirely consistent across all cohorts [28], they support the view that CTNNB1-mutated tumors may retain clinically actionable recurrence potential, thereby justifying a surveillance intensity higher than that of biologically low-risk NSMP.

Within the high-risk group, recurrence—if it occurs—is more likely to present as systemic or multifocal disease, and post-recurrence prognosis tends to be significantly worse. Assignment to this group may be considered in the presence of: (i) the p53abn subtype, irrespective of stage; (ii) stage III–IV disease, irrespective of molecular subtype; or (iii) adverse clinicopathological features such as sentinel lymph node macro- or micrometastases, substantial LVSI, or L1CAM overexpression, particularly within NSMP. In the NSMP subtype, a more aggressive biological phenotype—especially high-grade, ER-negative, or L1CAM-positive tumors—in combination with advanced anatomical stage may support high-risk assignment. In this group, the proposed surveillance goal is primarily rapid initiation of systemic therapy or enrollment in clinical trials, rather than routine detection of asymptomatic recurrence.

In cases where CTNNB1 testing is unavailable, classification within NSMP may reasonably be based on ER status, with the understanding that this approach is inherently surrogate and may lead to underestimation of risk in tumors that would otherwise meet criteria for biologically less favorable NSMP subgroups. The role of ER and L1CAM as modifiers should be interpreted cautiously, as their prognostic impact within NSMP remains heterogeneous across studies and has not yet been prospectively validated in the context of surveillance-oriented stratification.

### 4.3. Proposed Classification Algorithm

**Figure 1:**
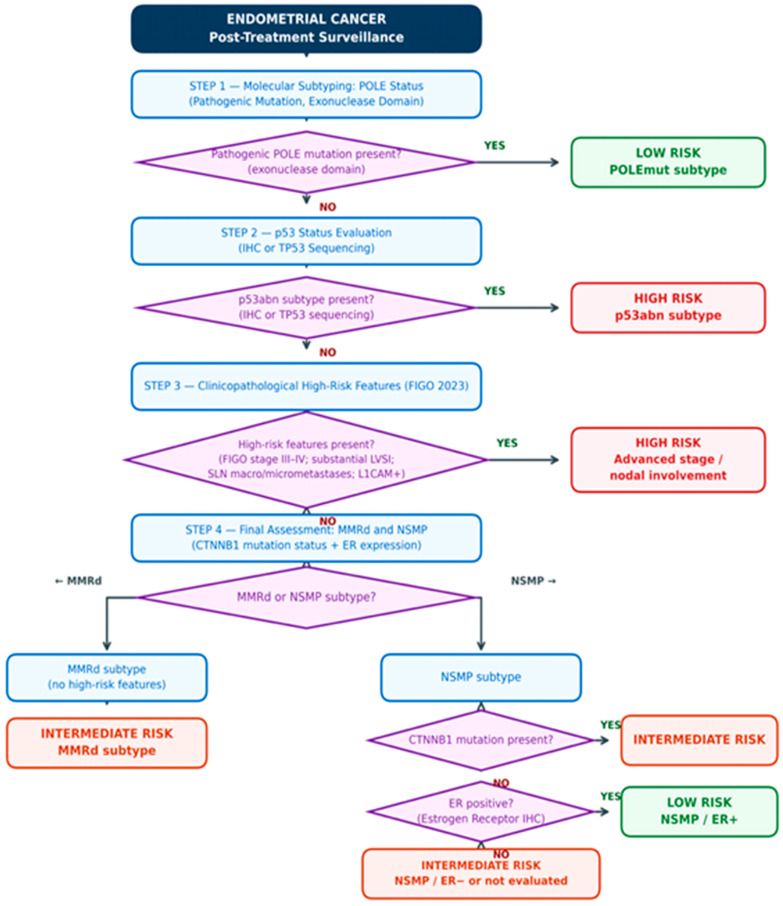
Proposed surveillance risk stratification algorithm for endometrial cancer patients following primary treatment. The algorithm follows a sequential molecular-to-clinicopathological logic: (1) identification of the ultra-favorable POLEmut group; (2) exclusion of the high-risk p53abn subtype; (3) assessment of clinicopathological high-risk features (FIGO 2023 stage III–IV, substantial LVSI, macro-/micrometastases in lymph nodes, L1CAM expression); and (4) final refinement of MMRd and NSMP cohorts using CTNNB1 mutation status and estrogen receptor (ER) expression. Risk groups are color-coded: green = low risk, orange = intermediate risk, red = high risk. Abbreviations: POLE = DNA polymerase epsilon (exonuclease domain); p53abn = abnormal p53 expression (IHC); IHC = immunohistochemistry; LVSI = lymphovascular space invasion; L1CAM = L1 cell adhesion molecule; MMRd = mismatch repair deficient; NSMP = no specific molecular profile; CTNNB1 = beta-catenin gene; ER = estrogen receptor; FIGO = International Federation of Gynecology and Obstetrics 2023.

The proposed classification algorithm for surveillance risk group assignment is illustrated in Figure 1. It follows a four-step sequential decision-making approach based on molecular subtype assessment followed by evaluation of clinicopathological factors that may modify recurrence risk. The algorithm assumes the availability of basic molecular classification, including assessment of POLE mutation status, MMR status, and p53 immunohistochemical phenotype.

In the first step, identification of POLEmut tumors would lead to low-risk assignment irrespective of stage and other clinicopathological features. In the second step, identification of p53abn would support high-risk assignment irrespective of stage. The third step would involve assessment of high-risk clinicopathological factors—stage III–IV, sentinel lymph node macro/micrometastases, substantial LVSI, and, within NSMP, L1CAM overexpression—the presence of which could support high-risk group assignment.

Within the NSMP subtype, assessment of CTNNB1 mutation status is proposed as the primary modifier. The presence of a CTNNB1 mutation supports assignment to the intermediate-risk group, even when anatomical low-risk criteria are otherwise fulfilled. When CTNNB1 mutation is absent or test results are unavailable, further classification may be based on ER status. In this context, patients with ER-positive NSMP presenting with low anatomical stage, the absence of CTNNB1 mutation, and absence of L1CAM overexpression may be considered for low-risk surveillance. Conversely, ER-negative NSMP in an analogous setting may reasonably be treated as intermediate risk.

The proposed algorithm is pragmatic in character and is not intended to replace prognostic systems used for adjuvant treatment selection. Rather, it is designed to suggest how surveillance intensity may be aligned with the biologically anticipated recurrence phenotype. In the absence of complete molecular classification, the surveillance strategy appropriately falls back on classical clinicopathological factors in accordance with current guidelines.

## 5. Proposed Surveillance Schedule

The proposed post-treatment surveillance schedule for each risk group is presented in Table 3.

These observations suggest that patterns of recurrence, and therefore optimal surveillance strategies, may be influenced by previous adjuvant treatment. However, this hypothesis requires prospective validation.

Clinical examination forms the cornerstone of surveillance in all three risk groups—a targeted history focused on symptoms of recurrence and a gynecological physical examination including speculum examination and bimanual pelvic assessment. The value of clinical examination in detecting symptomatic recurrence is well established, and data from the TOTEM trial confirm that extending surveillance to include routine laboratory tests and imaging does not improve overall survival [4,5]. Additional investigations should therefore be used selectively, based on individual clinical assessment and tumor biology.

The intensity and focus of the clinical examination differ across risk groups. In the intermediate-risk group (MMRd, NSMP with risk factors), where the expected recurrence phenotype is frequently locoregional, particular emphasis should be placed on a thorough pelvic examination—both speculum assessment for early vaginal vault changes and careful bimanual evaluation of the parametria, pelvic sidewalls, and accessible lymph nodes. The history should focus on locoregional symptoms: vaginal bleeding or discharge, pelvic pain, and lower limb edema. In the high-risk group (p53abn, stage III–IV), where systemic recurrences predominate, the history should encompass a broader spectrum of symptoms: abdominal pain, bloating, increased abdominal girth, dyspnea, cough, and unexplained weight loss. Physical examination should include abdominal palpation (ascites, masses) and assessment of performance status.

CA125 measurement is primarily justified in the intermediate- and high-risk groups, but in distinct clinical contexts. In the intermediate-risk group, CA125 may be measured regularly (every three to six months) in patients in whom it was elevated at baseline, as an adjunct marker for early detection of locoregional recurrence. In the high-risk group, a low threshold for regular CA125 testing (every three to four months in the first two years) reflects the need to monitor for systemic progression and facilitate prompt treatment initiation. The value of CA125 remains limited; sensitivity for detecting early recurrences is low, and false-positive results can generate unnecessary diagnostic workup and patient anxiety.

Imaging—pelvic MRI, abdominal and thoracic CT, PET-CT—is not recommended routinely in the low-risk group. The key difference between the intermediate- and high-risk groups concerns the type and purpose of imaging, reflecting the distinct expected recurrence phenotype.

In the intermediate-risk group (MMRd, NSMP with risk factors), the priority is detection of locoregional recurrence—vaginal, pelvic, or nodal—that may qualify for radical treatment (salvage radiotherapy, surgical resection) or, in the case of MMRd, for immunotherapy. In this context, selective use of pelvic MRI when clinical suspicion arises (abnormality on gynecological examination, pelvic pain, lower limb edema, rising CA125) has greater diagnostic value than routine whole-body CT. MRI offers superior soft tissue resolution and more precise assessment of the vaginal vault, parametria, and regional lymph nodes. Imaging in this group is targeted in nature—employed on specific clinical suspicion rather than as routine screening. A lower threshold for imaging should be considered in patients with ER-negative NSMP or NSMP with CTNNB1 mutation, given the more unpredictable clinical course in these subgroups.

In the high-risk group (p53abn, stage III–IV), the expected recurrence phenotype is systemic dissemination—pulmonary, hepatic, peritoneal, and distant nodal metastases. The surveillance goal in this group is not routine detection of asymptomatic recurrence, but rather prompt eligibility assessment for systemic therapy or clinical trials at the earliest sign of progression. Chest, abdominal, and pelvic CT may be considered on an individualized basis in the first two years after treatment (e.g., every six to twelve months), particularly in p53abn patients at stage I–II, where early detection of asymptomatic recurrence could potentially influence eligibility for systemic treatment. It should be emphasized, however, that the impact of routine imaging on overall survival in this group remains unproven, and the potential benefits must be weighed against costs, radiation exposure, and the risk of false-positive results. In advanced stages (III–IV) or in patients following adjuvant treatment, imaging should be performed based on clinical symptoms or rising CA125.

Routine vaginal vault cytology is not recommended in any risk group. Multiple lines of evidence indicate that its sensitivity for detecting locoregional recurrence is lower than that of clinical examination, and the current ESGO 2025 guidelines do not recommend it as a component of standard surveillance [8].

An important complementary element of the surveillance structure is patient education. Patients in all risk groups should be informed about symptoms requiring prompt medical contact, with the educational content tailored to the expected recurrence phenotype. In the intermediate-risk group, particular emphasis should be placed on locoregional symptoms: vaginal bleeding or discharge, pelvic pain, and lower limb edema. In the high-risk group, education should cover a broader spectrum of systemic symptoms: abdominal pain, bloating, increased abdominal girth, new changes in bowel habits, dyspnea, cough, and unexplained weight loss. This approach—referred to as symptom-triggered surveillance or patient-led surveillance—complements scheduled clinical visits and may shorten the interval between symptom onset and diagnosis, particularly in the low-risk group where visit frequency is deliberately limited. These standards are consistent with practices employed in national oncology models—including the Danish endometrial cancer surveillance guidelines—which explicitly incorporate patient education as an integral component of surveillance [9].

## 6. Discussion

### 6.1. Post-Treatment Surveillance and the Question of Clinical Value

The proposed framework stems from a fundamental reframing of the surveillance question. Rather than asking how often and how intensively patients should be monitored, we suggest that the more clinically productive question is which recurrences are worth detecting earlier, because doing so changes management. Within this framework, the value of surveillance is defined by the therapeutic modifiability of recurrence, rather than by detection frequency per se. This distinction has direct practical consequences: it supports de-escalation in groups where detected recurrences are unlikely to alter treatment, and targeted intensification where early identification enables curative-intent intervention or biologically informed systemic therapy.

The TOTEM trial, despite demonstrating no survival benefit from intensive surveillance and no improvement in health-related quality of life [4,5], was designed before molecular subtype was recognized as a determinant of recurrence biology. Its results therefore do not preclude benefit from molecularly stratified surveillance and should not be extrapolated to patient groups defined by molecular criteria.

Although quantitative estimates of survival benefit are not currently available, the framework assumes that its clinical impact would be primarily mediated through improved selection for salvage treatment and timely initiation of systemic therapy.

### 6.2. Molecular Subtype as a Determinant of Surveillance Value

The clinical heterogeneity of endometrial cancer recurrence—documented most comprehensively in the KImBer cohort [16] and corroborated by molecular analyses of the PORTEC-3 trial [13]—directly informs the proposed stratification logic. The critical surveillance implication is not simply that recurrence rates differ by subtype, but that the phenotype of recurrence differs in a therapeutically meaningful way. Locoregional relapses in MMRd and NSMP may be intercepted at a stage where curative-intent treatment remains feasible; systemic relapses in p53abn typically are not. Surveillance intensity should therefore reflect this asymmetry rather than baseline recurrence probability alone.

Post-recurrence survival differences across subtypes—ranging from 43 months in MMRd to 10 months in p53abn [16]—further support this argument. In groups where post-recurrence prognosis is more favorable, earlier detection may be more likely to create an opportunity for clinically meaningful intervention. Importantly, long-term data from PORTEC-3 confirm that molecular subtype differences in recurrence risk and treatment benefit persist beyond ten years of observation [15], reinforcing the rationale for sustained, biology-informed surveillance rather than uniform, time-limited surveillance.

### 6.3. The Particular Role of Immunotherapy in MMRd Surveillance

The emergence of effective immunotherapy for mismatch repair-deficient (MMRd) endometrial cancer substantially strengthens the rationale for attentive, biology-informed surveillance in this subgroup. In contrast to the pre-immunotherapy era, where treatment options for recurrent disease were limited, the availability of immune checkpoint inhibitors has introduced a clinically meaningful opportunity for durable disease control in selected patients.

Prospective clinical trials have demonstrated that PD-1 inhibitors, including dostarlimab and pembrolizumab, achieve substantial and durable responses in MMRd/MSI-H endometrial cancer, with objective response rates exceeding 40% and a considerable proportion of long-term responders [19,20]. These outcomes contrast with the more limited efficacy of systemic therapies in other molecular subtypes, particularly p53abn tumors, which are characterized by lower response rates and shorter survival following recurrence.

From a surveillance perspective, this subtype-specific therapeutic responsiveness has direct implications. In MMRd tumors, early detection of recurrence may enable timely initiation of immunotherapy at a lower disease burden, which in other solid tumors has been associated with improved treatment tolerability and potentially enhanced efficacy. Although prospective data specifically addressing this question in endometrial cancer are limited, the biological rationale is compelling and aligns with the observed patterns of recurrence in this subgroup, which are frequently locoregional and potentially clinically actionable.

Importantly, the clinical utility of this approach depends on the assumption that molecular characteristics of the primary tumor are preserved at recurrence. Available evidence suggests that concordance of MMR status between primary and recurrent endometrial cancer is generally high, supporting the use of initial molecular classification to guide surveillance strategy [21]. Nevertheless, prospective validation of this assumption remains warranted.

In contrast, in molecular subtypes with limited responsiveness to immunotherapy, particularly p53abn, the clinical benefit of early detection is less directly linked to therapeutic modification. In these patients, recurrence is more often systemic and associated with poorer outcomes, and the primary surveillance objective may shift toward facilitating rapid treatment initiation or clinical trial enrollment rather than early detection of asymptomatic disease. This divergence further supports a differentiated, biology-driven approach to post-treatment surveillance.

### 6.4. The Proposed Model in Relation to Existing Guidelines

The proposed surveillance framework differs from current ESGO–ESTRO–ESP and NCCN recommendations primarily in its explicit integration of molecular subtype, recurrence phenotype, and therapeutic modifiability as determinants of surveillance intensity. While contemporary guidelines have incorporated molecular classification into risk stratification and adjuvant treatment decision-making, their impact on post-treatment surveillance remains limited. In most cases, surveillance schedules continue to rely predominantly on clinicopathological risk groups, with relatively uniform recommendations within broad categories [8,29,30,31]. A structured comparison between the proposed framework and current ESGO–ESTRO–ESP 2025 and NCCN recommendations is presented in Table 4.

In contrast, the present model proposes a shift from stage-based to biology-driven surveillance, in which surveillance intensity is aligned not only with the probability of recurrence, but also with its expected clinical relevance. This distinction is particularly important in the context of molecular subtypes with divergent recurrence behavior. In MMRd and selected NSMP tumors, where recurrence is more frequently locoregional and potentially amenable to curative-intent treatment or immunotherapy, closer clinical surveillance may be justified. Conversely, in p53abn tumors, where recurrence is typically systemic and associated with limited therapeutic options, the value of early detection is less clearly linked to improved outcomes. In this context, surveillance strategies may reasonably prioritize rapid treatment initiation or clinical trial enrollment over the routine detection of asymptomatic disease.

While the guidelines emphasize risk stratification primarily for adjuvant treatment decisions, the present model extends this concept into the post-treatment setting, introducing therapeutic modifiability as a key determinant of surveillance value. Importantly, this approach does not advocate for increased overall surveillance intensity, but rather for its redistribution according to biological risk and clinical utility.

An important limitation of the proposed modifier-based stratification within the NSMP subtype relates to the heterogeneity of available evidence. The prognostic impact of L1CAM expression, although consistently associated with adverse outcomes in several cohorts, appears to be influenced by stage distribution and coexisting high-risk features, raising the question of whether it represents an independent driver of aggressive disease or a surrogate marker of unfavorable clinicopathological context. Similarly, the role of estrogen receptor (ER) expression within NSMP remains incompletely defined. Some analyses suggest a favorable prognostic effect, particularly in low-stage disease [28], and this association is supported by recent meta-analyses [32]; however, the magnitude and independence of this effect may vary depending on cohort composition and adjustment for clinicopathological variables. These inconsistencies highlight the ongoing uncertainty regarding the prognostic role of ER within NSMP tumors. These discrepancies indicate that the integration of ER and L1CAM into surveillance-oriented stratification should be interpreted cautiously and considered hypothesis-generating.

Although the proposed framework has not been prospectively validated, its potential clinical and health-economic implications can be conceptually estimated. Assuming that patients with POLEmut tumors (~5–10% of all endometrial cancers) and a subset of biologically low-risk NSMP (~20–25%) could safely undergo de-escalated surveillance, a substantial proportion of patients may require fewer surveillance visits and imaging procedures. A reduction in imaging frequency alone could translate into a meaningful decrease in the number of CT or MRI scans performed per 100 patients annually. These considerations are consistent with the findings of the TOTEM trial, which demonstrated that intensive, non-stratified surveillance does not improve overall survival or quality of life, while increasing healthcare costs [4,5]. In this context, a biology-driven redistribution of surveillance intensity may represent a more rational allocation of resources, focusing clinical attention on patient groups in whom earlier detection of recurrence is most likely to alter management.

At the same time, these estimates remain hypothetical and should be interpreted with caution. Prospective validation and formal health-economic modeling are required to quantify the impact of risk-adapted surveillance on recurrence detection rates, treatment outcomes, and resource utilization. Until such data are available, the proposed framework should be regarded as a conceptual model intended to inform future clinical research rather than a prescriptive clinical guideline.

### 6.5. Limitations of the Proposed Model

The most important limitation of the proposed framework is the absence of prospective validation. No randomized trial evaluating molecularly informed surveillance strategies has been conducted to date, and the model should therefore be regarded as a hypothesis-generating proposal rather than a practice-changing recommendation.

A second limitation is the variable availability of molecular diagnostics. Despite expanding access to molecular profiling, complete TCGA-based classification remains inaccessible in many healthcare systems. Data from Poland illustrate the extent of inter-institutional variability in this regard [33].

Third, the NSMP subtype remains internally heterogeneous, and the roles of ER, CTNNB1, and L1CAM as surveillance modifiers require further validation [22,23,28]. Fourth, prior adjuvant treatment may itself modify recurrence patterns—data from the KImBer cohort suggest that adjuvant radiotherapy shifts recurrence distribution toward distant sites [16]—implying that surveillance strategies may need to account for treatment history in addition to molecular subtype.

Finally, the role of hormone replacement therapy after endometrial cancer treatment lies beyond the primary scope of this model. Available data suggest no substantial increase in recurrence risk in selected early-stage patients, but molecularly stratified data remain limited [34,35,36,37].

### 6.6. Future Directions and Prospective Validation

The proposed surveillance framework should be considered a hypothesis-generating model that requires prospective validation. Future studies should evaluate whether molecularly informed surveillance improves clinically meaningful outcomes compared with conventional surveillance strategies. Prospective cohort studies or registry-based analyses could assess recurrence patterns within molecular subtypes in the context of contemporary adjuvant therapies, including immunotherapy and combined systemic approaches. In addition to overall survival, relevant endpoints should include time to recurrence detection, proportion of salvageable recurrences, time to initiation of systemic therapy, patient-reported quality of life, and health-economic outcomes. Ultimately, a randomized comparison between biology-driven surveillance and standard surveillance strategies may be required to determine whether molecularly stratified surveillance provides measurable clinical benefit.

## 7. Conclusions

A uniform post-treatment surveillance schedule for endometrial cancer, based exclusively on clinical stage and histological grade, does not reflect the biological heterogeneity of this disease as revealed by TCGA-based molecular classification. The proposed framework integrates molecular subtype, clinicopathological factors, and the expected recurrence phenotype into a three-tier surveillance stratification model.

The key conceptual shift is a redefinition of the surveillance goal: instead of asking how often patients should be monitored, the more relevant question is which recurrences are worth detecting early because doing so may change clinical management. Locoregional recurrences, which are more frequent in the MMRd and NSMP subtypes, may remain amenable to curative-intent treatment and therefore justify heightened clinical vigilance. By contrast, systemic recurrences, which are more typical of p53abn disease, more often require prompt initiation of systemic therapy or consideration for clinical trial enrollment.

Prospective cohort studies are needed to evaluate recurrence patterns across molecular subtypes in the era of contemporary adjuvant treatment, particularly following the introduction of immunotherapy into the first-line setting. Ultimately, a randomized trial comparing molecularly stratified surveillance with conventional clinicopathological surveillance strategies would be required, with endpoints including quality of life, healthcare costs, and survival.

Until such data become available, the proposed model should be regarded as a biologically grounded decision-making framework and a starting point for further validation.

## Figures and Tables

**Table 3:** Proposed post-treatment surveillance schedule for endometrial cancer patients according to risk group.

Parameter	Low Risk	Intermediate Risk	High Risk	Notes
Molecular–clinical profile	POLEmut; or NSMP: ER+, CTNNB1wt, L1CAM−, stage I, G1–2, MI < 50%, LVSI absent/focal	MMRd without high-risk features; NSMP CTNNB1mut; NSMP ER—at low stage	p53abn (any stage); stage III–IV (any subtype); macro/micro-nodal metastases; substantial LVSI; L1CAM+	CTNNB1—primary modifier within NSMP
Years 1–2	Every 6 months	Every 4 months	Every 3 months	Highest recurrence risk within first 24 months
Years 3–5	Every 12 months	Every 6 months	Every 4–6 months	Late recurrences possible in MMRd and NSMP
After 5 years	Discharge or primary care follow-up	Individualized follow-up (~12 months)	Individualized follow-up (6–12 months)	Further surveillance based on ongoing risk assessment
Clinical examination	History + gynecological examination	History + gynecological examination; emphasis on bimanual pelvic exam	History + gynecological + abdominal examination; assess performance status	Cornerstone of surveillance in all groups
Imaging	Not routinely indicated	Pelvic MRI if clinically suspected recurrence; lower threshold in CTNNB1mut or ER−NSMP	Individualized chest/abdomen/pelvis CT; low threshold at earliest sign of progression	Aim: rapid eligibility assessment for salvage treatment or systemic therapy
CA125	Not routinely	Consider every 3–6 months if elevated at baseline	Low threshold; every 3–4 months in years 1–2	Adjunct marker; limited sensitivity for early recurrence
Vaginal cytology	Not recommended	Not recommended	Not recommended	Per ESGO–ESTRO–ESP 2025 guidelines
**Patient education**	Locoregional symptoms (vaginal bleeding, pelvic pain, lower limb edema)	Locoregional symptoms (as low risk); heightened awareness of nodal disease	Systemic symptoms: abdominal pain, bloating, dyspnea, cough, weight loss	Symptom-triggered follow-up complements scheduled visits

Abbreviations: MI—myometrial invasion; LVSI—lymphovascular space invasion; ER—estrogen receptor; CA125—cancer antigen 125; CT—computed tomography; MRI—magnetic resonance imaging; ESGO—European Society of Gynaecological Oncology; ESTRO—European Society for Radiotherapy and Oncology; ESP—European Society of Pathology. Routine vaginal vault cytology is not recommended per ESGO–ESTRO–ESP 2025 guidelines. Visit frequencies are proposed ranges; final decisions should be individualized based on clinical assessment.

**Table 4:** Comparison of the proposed biology-driven surveillance framework with ESGO–ESTRO–ESP 2025 and NCCN recommendations.

Parameter	ESGO–ESTRO–ESP 2025 [8]	NCCN Guidelines †	Proposed Framework (This Study)
**A. Basis of risk stratification**			
**Primary criterion**	Clinicopathological stage with integration of molecular subtype	Clinicopathological risk factors; molecular classification not primary determinant	Molecular subtype as primary criterion; clinicopathological factors as secondary modifiers
**Number of risk groups**	Four (low/intermediate/high–intermediate/high)	Low vs. higher risk	Three tiers (low/intermediate/high); intermediate ESGO groups merged pragmatically
**Staging system**	FIGO 2023	FIGO 2009 †	FIGO 2023
**Molecular classification**	Recommended for all patients	Recommended, not mandatory	Preferred; fall back to clinicopathological criteria if unavailable
**B. Molecular subtype-specific considerations**			
**CTNNB1 (NSMP)**	Emerging prognostic marker; not integrated into surveillance	Not incorporated	Candidate primary modifier (pending prospective validation): CTNNB1mut → intermediate risk
**ER (NSMP)**	Not used in surveillance	Not incorporated	Secondary modifier (when CTNNB1 unavailable): ER+ → lower risk; ER− → intermediate risk
**L1CAM (NSMP)**	High-risk marker	Not incorporated	High-risk modifier; supports high-risk classification
**p53abn**	High-risk subtype	High-risk subtype	High-risk tier; focus on systemic therapy rather than early detection
**C. Surveillance schedule**			
**Years 1–2**	Every 3–6 months	Every 3–6 months	Proposed: 3 months (high); 4 months (intermediate); 6 months (low)
**Years 3–5**	Every 6–12 months	Every 6 months	Proposed: 4–6 months (high); 6 months (intermediate); 12 months (low)
**Beyond 5 years**	Not defined	Annual or discharge	Risk-adapted: extended in selected MMRd and higher-risk NSMP; de-escalation in POLEmut and low-risk NSMP
**D. Investigations**			
**Vaginal cytology**	Not recommended	Not recommended	Not recommended
**Routine imaging**	Not routine; symptom-triggered	Symptom- and risk-directed †	Not routine (low); symptom-triggered MRI (intermediate); individualized CT (high)
**CA125**	Not routine	Consider if elevated at baseline or serous histology †	Risk-adapted: regular in intermediate (if baseline elevated); low threshold in high-risk
**Imaging modality**	CT or MRI when indicated	CT when indicated †	MRI preferred for locoregional assessment; CT for systemic disease evaluation
**E. Conceptual goal of surveillance**			
**Surveillance goal**	Recurrence detection; symptom monitoring	Recurrence detection; late effects management	Risk-specific: salvage treatment opportunity (intermediate); systemic therapy initiation (high); reassurance and de-escalation (low)
**Therapeutic modifiability**	Not explicit (implicitly reflected in adjuvant treatment strategies)	Not explicit	Core principle: surveillance intensity linked to whether early detection changes management
**Immunotherapy context**	Acknowledged in treatment recommendations	Acknowledged in treatment recommendations	Integrated into surveillance rationale for MMRd subtype

† NCCN Clinical Practice Guidelines in Oncology: Uterine Neoplasms (current version). NCCN guidelines currently reference FIGO 2009 staging; adoption of FIGO 2023 is ongoing. NCCN recommends imaging based on symptoms and clinical risk assessment, not routinely; CA125 monitoring is recommended when initially elevated or in cases of serous histology. The proposed framework does not advocate increased visit frequency per se, but biologically informed redirection of surveillance intensity—a distinction from the uniform intensification evaluated in the TOTEM trial [4,5]. CTNNB1 status as a surveillance modifier is a candidate criterion pending prospective validation. Abbreviations: ESGO—European Society of Gynaecological Oncology; ESTRO—European Society for Radiotherapy and Oncology; ESP—European Society of Pathology; NCCN—National Comprehensive Cancer Network; NSMP—no specific molecular profile; MMRd—mismatch repair-deficient; POLEmut—pathogenic POLE mutation; p53abn—aberrant p53 expression; ER—estrogen receptor; L1CAM—L1 cell adhesion molecule; CTNNB1—β-catenin gene; MRI—magnetic resonance imaging; CT—computed tomography; CA125—cancer antigen 125.

## Data Availability

No new data were generated or analyzed in this study.
